# Retrospective analysis of the association between intraoperative magnesium sulfate infusion and postoperative acute kidney injury after major laparoscopic abdominal surgery

**DOI:** 10.1038/s41598-019-39106-4

**Published:** 2019-02-26

**Authors:** Tak Kyu Oh, Ah-Young Oh, Jung-Hee Ryu, Bon-Wook Koo, Yea Ji Lee, Sang-Hwan Do

**Affiliations:** 1 Department of Anesthesiology and Pain medicine, Seoul National University Bundang Hospital, Seoul, Korea; 2 Department of Anesthesiology and Pain Medicine, College of Medicine, Seoul National University, Seoul, Korea

## Abstract

Magnesium sulfate can be used as a co-adjuvant drug during the perioperative period and has multiple benefits. Recent evidence suggested that perioperative magnesium sulfate infusion may lower the risk of postoperative acute kidney injury (AKI). We investigated the association between intraoperative magnesium sulfate infusion and incidence of AKI after major laparoscopic abdominal surgery. We retrospectively analyzed the medical records of adult patients 20 years or older who underwent elective major laparoscopic abdominal surgery (>2 hours) between 2010 and 2016. We investigated the association between intraoperative magnesium sulfate infusion and the incidence of postoperative AKI until postoperative day (POD) 3 using a multivariable logistic regression analysis. We included 3,828 patients in this analysis; 357 patients (9.3%) received an intraoperative magnesium sulfate infusion and 186 patients (4.9%) developed postoperative AKI by POD 3. A multivariable logistic regression analysis showed that magnesium infusion was associated with a significant decrease (63%) in postoperative AKI (odds ratio, 0.37; 95% confidence interval, 0.14–0.94; *P* = 0.037). Our study suggested that intraoperative magnesium sulfate infusion is associated with a reduced risk of postoperative AKI until POD 3 for patients who underwent laparoscopic major abdominal surgery. Well-designed, prospective studies should be conducted to further substantiate these findings.

## Introduction

Acute kidney injury (AKI) is defined as a state in which renal function rapidly declines, and it is reported to affect 5.0–7.5% of all inpatients and up to 20% of patients admitted to the intensive care unit (ICU)^1,2^. Furthermore, 40% of all AKI cases are known to develop postoperatively^3^, which aggravates recovery^4^ and increases hospital mortality and costs^5^. Therefore, identifying and preventing the risk factors for postoperative AKI are pressing tasks during perioperative patient management^6,7^.

Magnesium sulfate can be used as a co-adjuvant drug during the perioperative period^8^ because of its multiple benefits, such as improving postoperative pain^9^, potentiating intraoperative muscle relaxation^10^, and preventing postoperative nausea and vomiting^11,12^. Therefore, the importance of intraoperative magnesium infusion is more pronounced in the field of pain and anesthesia^13^. Recently, it has been hypothesized that magnesium replacement can reduce kidney injury^14^ because of the following reasons: a lower level of serum magnesium may accelerate renal dysfunction by inducing hyperphosphatemia in patients with renal dysfunction^15^ and magnesium sulfate has protective effects against lipid peroxidation in cellular membranes^16^. Lipid peroxidation is known to be a critical reactive oxygen pathway that induces ischemic tissue injury in AKI^17^. Therefore, magnesium sulfate administration has been studied in diabetic rats and was found to prevent diabetic nephropathy^18^. Furthermore, a recent study reported that premedication of intravenous magnesium lowered cisplatin-induced nephrotoxicity in cancer patients^19^. However, the renoprotective effects of magnesium sulfate remain controversial, and there is insufficient evidence to support its efficacy. In particular, the association between magnesium sulfate infusion during the perioperative period and postoperative AKI remains largely unknown.

Therefore, this study investigated the association between magnesium sulfate infusion during surgery and postoperative AKI for patients who underwent laparoscopic major abdominal surgery.

## Results

**Figure 1:**
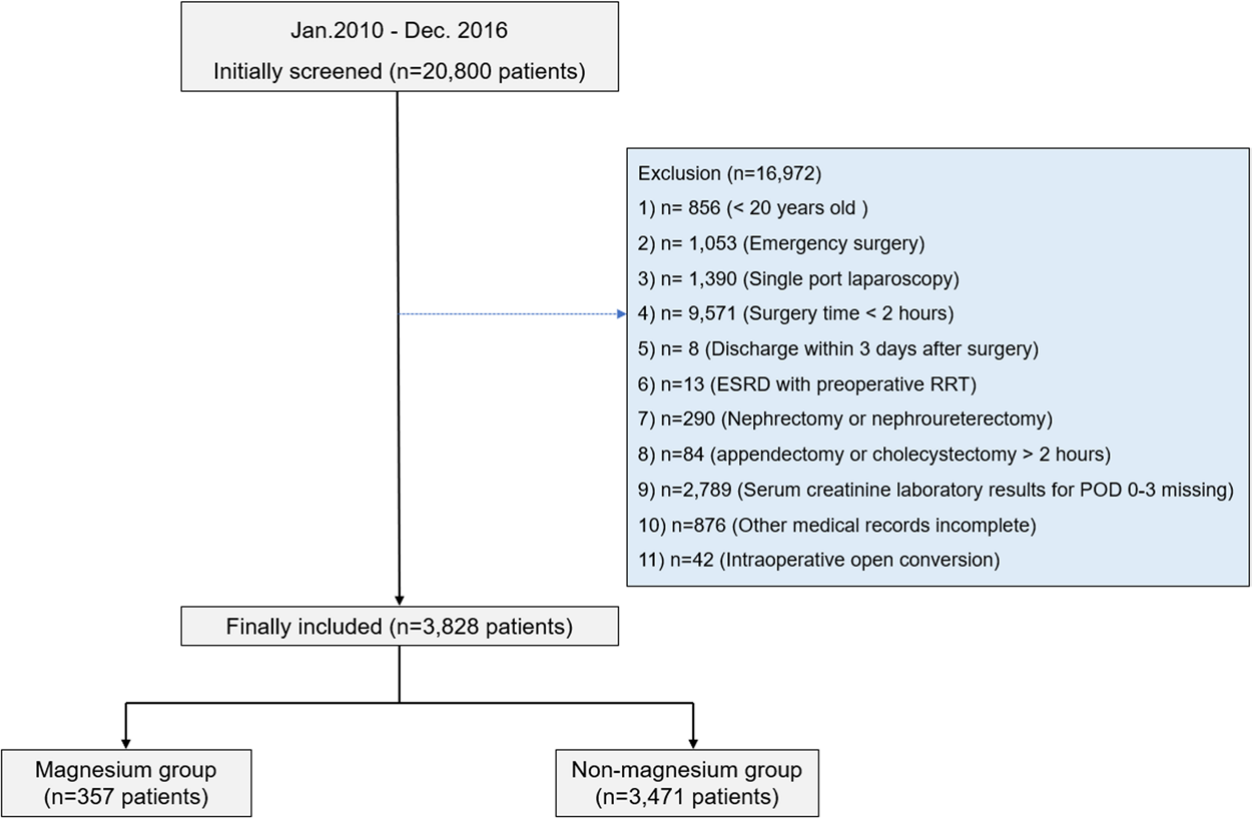
Flow chart for patient selection ESRD, end-stage renal disease; RRT, renal replacement therapy; AKI, acute kidney injury.

A total of 20,800 laparoscopic abdominal surgeries were performed at Seoul National University Bundang Hospital (SNUBH) between January 1, 2010 and December 30, 2016. We sequentially excluded the following cases: patients younger than 20 years (856); emergency surgery (1,053); single port laparoscopy (1,390); surgeries lasting less than 2 hours (9,571); discharged before POD 3 (8); ESRD patients (13); nephrectomy or nephroureterectomy (290); appendectomy or cholecystectomy requiring more than 2 hours (84); missing serum creatinine laboratory results for POD 0-3 (2,789); other medical records incomplete (876); and intraoperative open conversion (42). As a result, 3,828 patients who underwent a major laparoscopic abdominal surgery were included in the final analysis, and a total of 357 patients (9.3%) received intraoperative magnesium sulfate infusion (Fig. 1).

**Figure 2:**
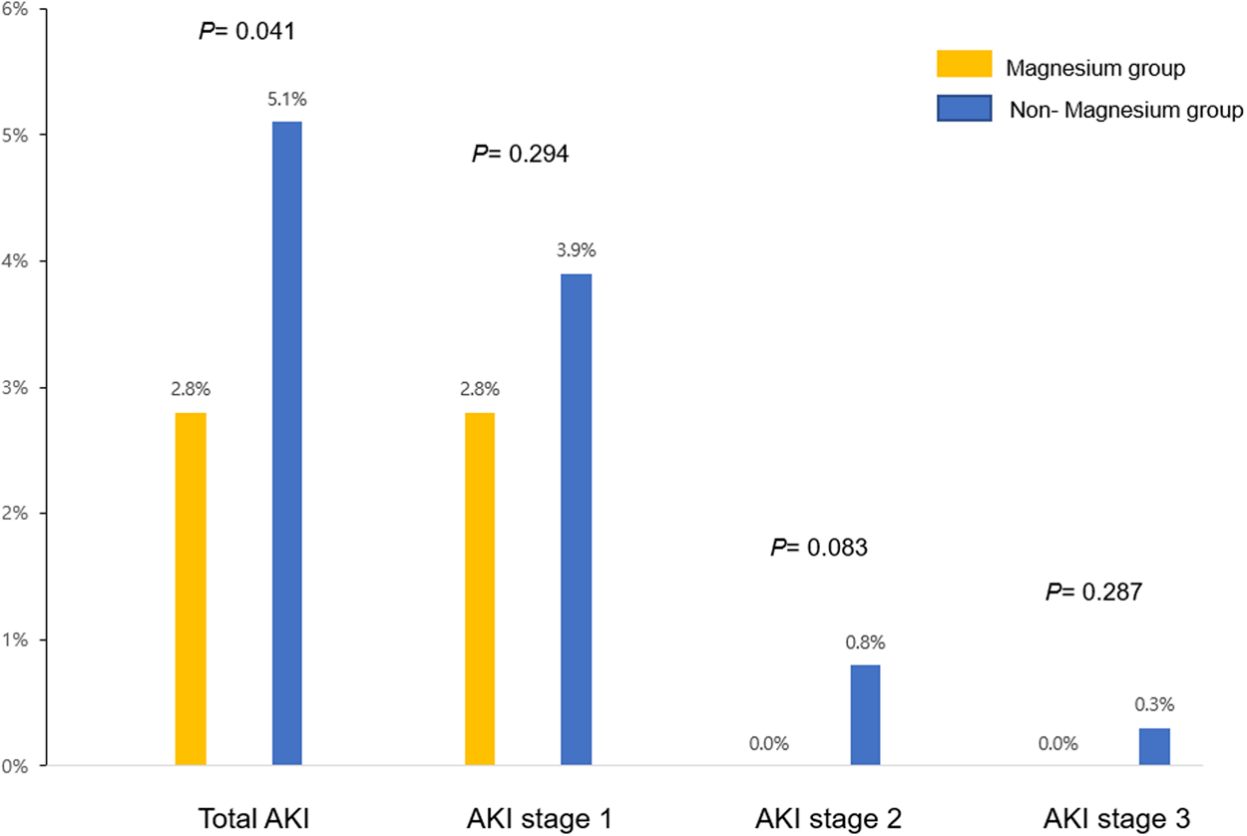
Probabilities of postoperative AKI development for the magnesium and non-magnesium groups AKI, acute kidney injury.

Table 1 compares the baseline characteristics and total AKI between the magnesium and non-magnesium groups. AKI incidents on POD 0-3 in the magnesium group were as follows: 10/357 (2.8%) for stage 1 AKI; 0 (0.0%) for stage 2 AKI; 0 (0.0%) for stage 3 AKI; and 10/357 (2.8%) for total AKI. AKI incidents on POD 0-3 in the non-magnesium group were as follows: 136/3,471 (3.9%) for stage 1 AKI; 29/3,471 (0.8%) for stage 2 AKI; 11/3,471 (0.3%) for stage 3 AKI; and 176/3,471 (5.1%) for total AKI (Fig. 2). The hospital length of stay for patients who experienced AKI on POD 0-3 was longer than that for patients without AKI (mean, 22.2 days; standard deviation [SD] 28.5; AKI group stay, 10.8 days; SD, 7.8; *P* < 0.001).

**Table 1:** Comparison between magnesium group and non-magnesium group for baseline characteristics.

Variables	Mg group	Non-Mg group	*P*-value
	n = 357	n = 3,471	
Age, yr	60.0 (13.4)	60.8 (13.0)	0.262
Sex: male	219 (61.3%)	2,208 (63.6%)	0.397
Body mass index, kg m^−2^	23.8 (3.5)	24.1 (3.5)	0.113
Preoperative comorbidities			
ASA physical status			0.052
1	140 (39.2%)	1,297 (37.4%)	
2	189 (52.9%)	1,996 (57.5%)	
3 + 4	28 (7.8%)	178 (5.1%)	
Diagnosis of cancer	296 (82.9%)	2,910 (83.8%)	0.652
Hypertension	127 (35.6%)	1,125 (32.4%)	0.225
Diabetes mellitus	57 (16.0%)	560 (16.1%)	0.935
Ischemic heart disease	34 (9.5%)	188 (5.4%)	0.002
Cerebrovascular disease	21 (5.9%)	113 (3.3%)	0.010
Dyslipidemia	12 (3.4%)	192 (5.5%)	0.082
Preoperative eGFR^a^, mL min^−1^ 1.73 m^−2^			0.863
≥90	207 (58.0%)	2,029 (58.5%)	
<90	150 (42.0%)	1,442 (41.5%)	
Operative Characteristics			
Surgery time, min	213.7 (92.6)	204.2 (76.2)	0.029
Postoperative ICU admission	8 (2.2%)	70 (2.0%)	0.845
Length of hospital stay, day	11.0 (6.3)	11.3 (10.4)	0.520
Staff anesthesiologists			<0.001
A	303 (72.0%)	118 (28.0%)	
Other anesthesiologists	54 (1.6%)	3,353 (98.4%)	
Potential risk of postoperative AKI on POD 0-3			
MBP < 60 mmHg during surgery over 1 min	160 (44.8%)	197 (55.2%)	0.003
Intraoperative vasopressor infusion	20 (5.6%)	48 (1.4%)	<0.001
Antibiotics or antiviral drug^b^ use	10 (2.8%)	182 (5.2%)	0.044
Radiocontrast use	16 (4.5%)	144 (4.1%)	0.765
H_2_ Antagonist or PPI use	330 (92.4%)	3,173 (91.4%)	0.509
Hydroxyethyl starch use	73 (9.6%)	687 (19.8%)	0.767
Non-steroidal anti-inflammatory drug use	80 (22.4%)	743 (21.4%)	0.660
Exposure of anemia (hemoglobin <10 mg dL^−1^)	31 (8.7%)	257 (7.4%)	0.383
Acute kidney injury on POD 0-3			
Stage 1	10 (2.8%)	136 (3.9%)	0.294
Stage 2	0 (0.0%)	29 (0.8%)	0.083
Stage 3	0 (0.0%)	11 (0.3%)	0.287
Total	10 (2.8%)	176 (5.1%)	0.041

Preoperative eGFR^a^ (mL min^−1^ 1.73 m^−2^): 186 × (Creatinine)^−1.154^ × (Age)^−0.203^ × (0.742 if female).

Antibiotics or antiviral drug^b^ includes vancomycin, cephalosporin, aminoglycoside, Rifampin, Acyclovir, and sulfonamide.

SD, standard deviation; ASA, American Society of Anesthesiologists; eGFR, estimated glomerular filtration rate; ICU, intensive care unit; AKI, acute kidney injury; POD, postoperative day; MBP, mean blood pressure; PPI, proton pump inhibitor; RRT, renal replacement therapy.

### Intraoperative magnesium sulfate infusion and risk of AKI

Tables 2 and 3 shows the results of the univariable and multivariable logistic regression analyses, which were conducted to identify the factors associated with postoperative AKI on POD 0-3 after major laparoscopic abdominal surgery. In the multivariable logistic model containing the covariates with *P* < 0.1 in the univariate logistic model, intraoperative magnesium infusion was significantly associated with a 63% lower incidence of postoperative AKI on POD 0-3 (odds ratio [OR], 0.37; 95% confidence interval [CI], 0.14–0.94; *P* = 0.037).

**Table 2:** Univariable logistic regression analysis for occurrence of postoperative acute kidney injury after laparoscopic major abdominal surgery in total patients.

Variables		Univariable model	
		Odds ratio (95% CI)	*P*-value
**Sex: Male (vs Female)**		1.52 (1.09–2.10)	**0.013**
**Age, year**		1.02 (1.01–1.03)	<**0.001**
**Body mass index, kg m** ^**−2**^		0.96 (0.92–1.00)	**0.066**
**Preoperative ASA physical status**	1	1	**(**<**0.001)**
	2	2.29 (1.56–3.36)	<0.001
	3 + 4	7.64 (4.63–12.61)	<0.001
**Preoperative eGFR** ^**a**^ **, mL min** ^**−1**^ **1.73 m** ^**−2**^	≥90	1	
	<90	0.69 (0.51, 0.94)	**0.020**
**Surgery time, min**		1.01 (1.01–1.01)	<**0.001**
Total venous intravenous anesthesia		0.70 (0.46–1.09)	0.116
**Staff anesthesiologist A (vs other anesthesiologists)**		**0.70 (0.41, 1.05)**	**0.092**
**Intraoperative magnesium sulfate use**		0.54 (0.28–1.03)	**0.061**
**Diagnosis of cancer**		0.39 (0.29–0.54)	<**0.001**
**Hypertension**		1.63 (1.21–2.19)	**0.001**
Diabetes mellitus		0.96 (0.64–1.44)	0.841
**Ischemic heart disease**		2.18 (1.35–3.51)	**0.001**
**Cerebrovascular disease**		1.80 (0.95–3.39)	**0.070**
Dyslipidemia		0.79 (0.38–1.63)	0.523
Potential risk of postoperative AKI on POD 0-3			
MBP < 60 mmHg during surgery over 1 min		1.04 (0.77, 1.40)	0.801
**Intraoperative vasopressor infusion**		11.28 (6.66, 19.09)	<**0.001**
Antibiotics or antiviral drug^b^ use		3.58 (2.31–5.54)	<**0.001**
H_2_ Antagonist or PPI use		0.92 (0.55–1.53)	0.745
**Radiocontrast**		3.01 (1.84–4.94)	<**0.001**
Non-steroidal anti-inflammatory drugs		1.21 (0.86–1.71)	0.272
**Hydroxyethyl starch use**		4.57 (3.38–6.16)	<**0.001**
**Exposure of anemia (hemoglobin <10 g dL**^**−1**^**)**		4.62 (3.23–6.60)	**<0.001**
**Year of surgery**	2010–2012	1	**(<0.001)**
	2013–2014	0.49 (0.33, 0.73)	**<**0.001
	2015–2016	0.43 (0.30, 0.60)	**<**0.001

Covariates of *P* < 0.1 in bold font were included in final multivariable logistic regression model.

Preoperative eGFR^a^ (mL min^−1^ 1.73 m^−2^): 186 × (Creatinine)^−1.154^ × (Age)^−0.203^ × (0.742 if female).

Antibiotics or antiviral drug^b^ includes vancomycin, cephalosporin, aminoglycoside, Rifampin, Acyclovir, and sulfonamide.

**Table 3:** Multivariable logistic regression analysis for occurrence of postoperative acute kidney injury after laparoscopic major abdominal surgery in total patients.

Variables		Multivariable model	
		Odds ratio (95% CI)	*P*-value
Sex: male (vs female)		1.47 (1.02, 2.11)	0.038
Age, yr		1.01 (0.99, 1.022)	0.242
Body mass index, kg m^−2^		0.98 (0.94, 1.03)	0.408
Preoperative ASA physical status	1	1	(**<**0.001)
	2	1.73 (1.09, 2.73)	0.019
	3 + 4	4.20 (2.18, 8.10)	**<**0.001
Preoperative eGFR^a^, mL min^−1^ 1.73 m^−2^	≥90	1	
	**<**90	0.49 (0.35, 0.71)	**<**0.001
Surgery time, min		1.00 (1.00, 1.01)	**<**0.001
**Intraoperative magnesium sulfate use**		0.37 (0.14, 0.94)	**0.037**
Staff anesthesiologist A (vs other anesthesiologists)		0.68 (0.31, 1.50)	0.340
Diagnosis of cancer		0.43 (0.30, 0.63)	**<**0.001
Hypertension		1.15 (0.79, 1.67)	0.476
Ischemic heart disease		1.17 (0.66, 2.07)	0.586
Cerebrovascular disease		0.99 (0.46, 2.14)	0.984
Potential risk of postoperative AKI on POD 0-3			
Intraoperative vasopressor infusion		4.19 (2.15, 8.16)	**<**0.001
Antibiotics or Antiviral drug use		2.18 (1.31, 3.63)	0.003
Radiocontrast use		1.83 (1.03, 3.27)	0.041
Hydroxyethyl starch use		1.79 (1.24, 2.58)	0.002
Exposure of anemia (hemoglobin **<**10 g dL^−1^)		2.00 (1.30, 3.07)	0.002
Year of surgery	2010–2012	1	(0.006)
	2013–2014	0.65 (0.42, 1.00)	0.049
	2015–2016	0.54 (0.36, 0.80)	0.002

Covariates of *P* < 0.1 in bold font were included in final multivariable logistic regression model.

Hosmer-Lemeshow statistics (Chi-square: 3.65, df: 8, *P* = 0.888).

Preoperative eGFR^a^ (mL min^−1^ 1.73 m^−2^): 186 × (Creatinine)^−1.154^ × (Age)^−0.203^ × (0.742 if female).

Antibiotics or antiviral drug^b^ includes vancomycin, cephalosporin, aminoglycoside, Rifampin, Acyclovir, and sulfonamide.

## Discussion

This study suggested that intraoperative magnesium sulfate infusion is associated with a lower incidence of AKI on POD 0-3 after a major laparoscopic abdominal surgery. Our findings are meaningful because this study only included laparoscopic surgical procedures, which are increasingly becoming more popular because of their ability to facilitate recovery^20^; other potential risk factors (e.g., antibiotics, radiocontrast, anemia) that may be associated with AKI until POD 3 were also included in the analysis.

The significant association between magnesium sulfate infusion and a lower risk of postoperative AKI may be attributable to several factors. First, the renoprotective effects of magnesium against hypoxic renal tissue injury, which has been suggested based on *in vitro* and animal studies, have had a role in the lower risk^16,17^. Previous studies showed that magnesium sulfate was associated with protection against oxidative damage from acute renal ischemia^16,17^. Based on this assumption, magnesium was reported to be associated with renoprotective effects against cisplatin-induced AKI^19,21^, contrast-induced AKI^22^, and diabetic nephropathic kidney injury^18^. In our study, patients were exposed to several agents that could induce nephrotoxicity on POD 0-3, including non-steroidal anti-inflammatory drugs, radiocontrast, antibiotics or antiviral drugs, and hypotension or anemia. The nephrotoxicity of these clinical events is associated with oxidative renal injury, and the infusion of magnesium sulfate may protect the renal system following the induction of nephrotoxicity on POD 0-3.

According to an animal study, magnesium is known to have anti-inflammatory effects^23^. A recent *in vivo* study showed that magnesium sulfate attenuated the inflammatory response of the placenta perfused with lipopolysaccharide^24^. Because inflammation is related to the pathophysiology of AKI^25^, the potential anti-inflammatory effects of magnesium sulfate might have been associated with postoperative AKI in this study. Although recent studies reported potential renoprotective effects of magnesium sulfate^18,26^, this is still a controversial issue, and further prospective clinical trials should be performed^14^.

Interestingly, this study found other potential risk factors for postoperative AKI, such as intraoperative vasopressor infusion, antibiotics or antiviral drug use, radiocontrast use, hydroxyethyl starch use, and exposure of anemia. Antibiotics or antiviral drug use, radiocontrast, anemia, and hydroxyethyl starch might have a role in nephrotoxicity itself, as reported in previous studies^27^. Vasopressor infusion, antiviral drugs, or hydroxyethyl starch could be used for patients who are critically ill during the perioperative period because of sepsis or shock. With perioperative shock or sepsis, postoperative AKI might occur frequently^28,29^. Therefore, these factors associated with postoperative AKI should be further interpreted.

This study has a clinical impact because it can be a useful reference for future prospective, randomized trials in the perioperative setting. Scientifically, sample size estimation is necessary to show the statistical significance of the results, if any, to avoid the recruitment of an excessively large sample cohort^30^. For example, with an objective of a 50% reduction in the incidence of postoperative AKI with a 0.05 chance of type 1 error and 80% power, using an incidence of 5.1% (observed in the total patients in this study), 848 patients in the magnesium group and the non-magnesium group are needed. To our knowledge, there was no background study that evaluated the effects of intraoperative magnesium sulfate infusion on the occurrence of postoperative AKI in the perioperative setting. Therefore, our results can contribute to the design of future prospective trial.

This study had a few limitations. First, there was a possibility of selection bias due to the retrospective nature of our study design. Second, the results may not be generalizable because this study was conducted at a single center. Third, we only used serum creatinine as the criterion for AKI diagnosis because we could not accurately measure the hourly urine output of the patients. Therefore, a considerable number of patients without serum creatinine data on POD 0-3 were excluded from this study. Finally, because we intended to relatively analyze the homogenous surgical population, many patients were excluded from this analysis, which limited its generalizability to other surgical populations. Nevertheless, this analysis is meaningful because it is the first human study performed in the perioperative setting that suggested the potential renoprotective effects of magnesium sulfate against postoperative AKI.

In conclusion, this study suggested that intraoperative magnesium sulfate infusion is associated with the reduced potential risk of postoperative AKI until POD 3 for patients who underwent laparoscopic major abdominal surgery. In the future, well-designed prospective studies should be conducted to further substantiate these findings.

## Methods

This study was a retrospective cohort study that was approved by the Institutional Review Board (IRB) of Seoul National University Bundang Hospital (SNUBH) (approval number: B-1803/459-105; approval date: 2018.03.12). The informed consent requirement was waived by the IRB due to the retrospective nature of this study, and this work adhered to the applicable STROBE guidelines.

### Patients

The medical records of patients 20 years or older who underwent elective laparoscopic major abdominal surgery at SNUBH between January 1, 2010 and December 31, 2016 were analyzed. Major laparoscopic abdominal surgery was defined as a surgical procedure involving laparoscopy-guided resection of an intraperitoneal organ that lasted more than 2 hours. We included only laparoscopic procedures for two reasons. First, the performance of laparoscopic procedures has been continuously increasing, resulting in these procedures becoming the most common surgical technique^31^. Second, the reduction of surgical trauma and carbon dioxide pneumoperitoneum during laparoscopic surgery is known to attenuate immune-mediated inflammatory responses, which could affect the occurrence of AKI^32,33^.

Even when the surgery involved resection of the intraperitoneal organ that lasted for more than 2 hours, we excluded the following patients: patients who underwent emergency surgery; patients who underwent single-port laparoscopy due to the possibility of fewer inflammatory responses during surgery compared to multi-port laparoscopy; patients discharged by postoperative day (POD) 3; end-stage renal disease (ESRD) patients who underwent renal replacement therapy (RRT) during the preoperative period; patients who underwent nephrectomy or nephroureterectomy, which are surgical procedures that may affect renal functions; patients who underwent simple appendectomy or cholecystectomy; patients with incomplete medical records, including serum creatinine data; and patients who underwent intraoperative open conversion.

### Laparoscopic major abdominal surgery at SNUBH

At SNUBH, experienced surgical teams proficiently resected major intraperitoneal organs (liver^34^, stomach^35^, colorectal surgery^36^) during the study period. Anesthetic management generally involved balanced anesthesia using desflurane and remifentanil or total intravenous anesthesia using propofol and remifentanil.

### Magnesium sulfate infusion during surgery (exposure group)

Magnesium sulfate infusion for anesthesia was used mainly by S. H. Do (defined as anesthesiologist A in Table 1) at SNUBH for laparoscopic surgery with the intention of improvement of surgical space conditions and less postoperative pain^37^. In the operating room, magnesium sulfate was infused beginning from the induction of anesthesia until the end of surgery. When administering magnesium sulfate, a mixture of 50 mg kg^−1^ of magnesium sulfate in 100 mL isotonic saline was infused over 15 minutes during the induction of anesthesia, and the infusion rate was adjusted throughout the surgery using the reference rate of 15 mg kg^−1^ h^−1^ based on the patient’s vital signs. If there was a complication due to magnesium sulfate infusion during surgery, such as hypotension, then adequate hydration or the use of a vasopressor was performed by the anesthesiologist. During the study period, there was no reported severe complication of magnesium sulfate infusion. We defined the magnesium sulfate group as the patients who received magnesium sulfate during surgery; the other patients were defined as the non-magnesium group.

#### Diagnosis of postoperative AKI on POD 0-3 (dependent variable)

Postoperative AKI was diagnosed per the criteria and grading suggested by the Kidney Disease: Improving Global Outcomes (KDIGO)^38^. However, considering the varying periods of urinary catheter use across laparoscopic surgical procedures, only serum creatinine (not urine output) was used for a more accurate diagnosis of AKI. At SNUBH, serum creatinine (mg dL^−1^) is measured within 1 month of scheduled surgery for all patients who are scheduled to undergo an elective surgery. This measurement was defined as preoperative serum creatinine (that is, baseline serum creatinine).

AKI stage 1 was defined as a serum creatinine level exceeding 0.3 mg/dL or an increase in serum creatinine by 1.5- to 1.9-times that of the preoperative level. AKI stage 2 was defined as an increase in serum creatinine by 2.0- to 2.9-times that of the preoperative level. AKI stage 3 was defined as serum creatinine level exceeding 4.0 mg/dL, an increase in serum creatinine by more than three-times that of the preoperative level, or a new round of RRT within 48 hours. These assessments and the diagnosis of postoperative AKI were performed from POD 0 to 3.

### Covariates

Patients’ demographic, clinical, and surgery-related information were collected for analysis. Preoperative kidney function was assessed based on the estimated glomerular filtration rate (eGFR) (mL min^−1^ 1.73 m^−2^), which was calculated using the Modification of Diet in Renal Disease calculation^39^: 186 × (preoperative serum creatinine)^−1.154^ × (age)^−0.203^ × (0.742 if female). The performance of surgery by a staff anesthesiologist (anesthesiologist A and others) was also included as a covariate. In addition, we collected the following data, which may represent potential risk factors for postoperative AKI on POD 0-3: antibiotics or antiviral drug use (vancomycin, cephalosporin, aminoglycoside, rifampin, acyclovir, and sulfonamide); radiocontrast use; histamine 2 receptor antagonist or proton pump inhibitor use; development of intraoperative hypotension (mean blood pressure <60 mmHg over 1 minute); hydroxyethyl starch use; non-steroidal anti-inflammatory drug use; vasopressor use; and development of anemia (hemoglobin <10 g dL^−1^).

### Study endpoint

The purpose of this study was to examine whether the incidence of AKI was reduced during the 3 days after major laparoscopic abdominal surgery when magnesium sulfate was used for anesthetic management compared to when magnesium sulfate infusion was not used.

### Statistical analysis

Patients’ baseline characteristics are presented as the number and percentage or mean and SD. Student’s *t*-test and Chi-squared test were used for continuous and categorical variables, respectively, to compare the magnesium and non-magnesium groups. We first performed the univariable logistic regression analysis to identify factors that may be associated with postoperative AKI. Next, after confirming that the covariates that satisfy *P* < 0.1 during the univariable logistic regression analysis had no problems with multicollinearity (variance inflation factors between variables <2.0), a final multivariable logistic regression analysis was performed with these covariates. The goodness of fit for each final multivariable logistic model was tested using the Hosmer and Lemeshow tests. Because AKI is related to preoperative kidney dysfunction^40^, we tested the interaction of intraoperative magnesium infusion with preoperative eGFR resulting in the occurrence of AKI to determine the necessity for a subgroup analysis. After confirming that there was no significant interaction between intraoperative magnesium infusion and preoperative eGFR resulting in the occurrence of AKI, we did not perform a subgroup analysis.

To detect a 3% difference in the incidence of postoperative AKI between the magnesium group and non-magnesium group with a 0.05 chance of a type 1 error and 80% power, a total of 3,839 patients (magnesium group, 349; non-magnesium group, 3,490) were needed. All statistical analyses were performed using IBM SPSS 24.0 software (IBM Corp., Armonk, NY); statistical significance was set at *P* < 0.05.

## Data Availability

The datasets used and/or analyzed during the current study are available from the corresponding author upon reasonable request.
